# Multi-omics signatures of chronic inflammation across immune-related disease states

**DOI:** 10.3389/fimmu.2026.1753156

**Published:** 2026-02-12

**Authors:** Hui Li, Xiaolin Xie, Lirui Tang, Chuanben Chen, Jinluan Li

**Affiliations:** Department of Radiation Oncology, Clinical Oncology School of Fujian Medical University, Fujian Cancer Hospital, Fujian, China

**Keywords:** chronic inflammation, competing risks, deep learning, immune cell communication, *in vitro* validation, multi-omics, stacking ensemble

## Abstract

**Introduction:**

Chronic inflammation and immune cell communication underpin a wide range of chronic diseases, yet population-scale maps integrating systemic inflammatory, metabolic and proteomic signals across multiple disease states are scarce.

**Methods:**

Using UK Biobank, we classified participants into six baseline groups—healthy controls, cancer, autoimmune, infectious, metabolic diseases, and multiple comorbidities. We profiled clinical and hematological indices, NMR-based metabolites and Olink proteomics, and trained four multi-class deep learning models (clinical/inflammatory only; +NMR; +Olink; three-tower multi-omics) with 10-fold cross-validation. Out-of-fold predicted probabilities were combined in a stacking meta-model to derive machine-learning risk scores for “any chronic disease.” Shapley value analyses were used to identify key features reflecting systemic immune and metabolic communication. Cause-specific cumulative incidence and Fine–Gray competing-risks models evaluated associations between these risk scores and cancer-related and non-cancer mortality, adjusting for conventional risk factors. To provide biological validation of model-prioritized immune mediators (BAFF [TNFSF13B], GDF15, IL-15 and CD276), we performed *in vitro* stimulation of healthy-donor PBMCs by ELISA, flow cytometry, and qPCR.

**Results:**

We observed pronounced and pathway-specific heterogeneity of inflammatory markers, lipid-related metabolites and immune–inflammatory proteins across disease groups. Omics-augmented deep learning models outperformed the clinical-only model, and the stacking ensemble achieved the best accuracy, macro-F1 and multi-class AUC. Machine-learning–derived risk scores showed monotonic gradients in cancer and other-cause death and remained independently associated with several cause-specific outcomes. *In vitro* validation supported myeloid inflammatory inducibility of model-highlighted mediators.

**Conclusions:**

By integrating multi-omics deep learning with competing-risks modelling, this study decodes population-level immune–metabolic communication patterns across chronic disease states, linking shared inflammatory and proteomic signatures to long-term mortality and providing a quantitative framework to support future, mechanism-focused and immunologically informed risk stratification.

## 1 Introduction

Immune-related chronic diseases, including cancer, autoimmune disorders, infection-prone conditions and metabolic disease, account for a growing share of global morbidity and premature mortality in ageing societies (1–5). At the patient level, these entities frequently cluster as multimorbidity, driven by excess adiposity, lifestyle exposures and social deprivation, and they impose a disproportionate burden on health-care systems (6). Large population-based cohorts and mechanistic studies now converge on chronic low-grade inflammation, dysregulated lipid and energy metabolism, and sustained immune activation as shared biological substrates linking these conditions (7–9). Recent high-throughput plasma proteomic and metabolomic atlases further demonstrate that coordinated inflammatory and metabolic signatures can predict a wide range of future disease events and all-cause mortality, often years before clinical diagnosis (10–12). However, how these systemic signatures map onto clinically recognisable clusters of immune-related disease states in the general population remains incompletely understood (12–14).

Despite rapid progress in population-scale omics profiling, most existing work has focused on predicting single endpoints—such as cardiovascular disease, type 2 diabetes, dementia or all-cause mortality—rather than mapping the broader landscape of immune-related multimorbidity (15–18). Large prospective cohorts have demonstrated that plasma metabolomic and proteomic signatures add substantial predictive value beyond traditional risk factors for mortality and cardiometabolic events, and can even approximate biological age and longevity risk (19, 20). However, these studies typically model one disease at a time, collapse heterogeneous immune and metabolic conditions into composite outcomes, or concentrate on organ-specific traits, thereby overlooking how shared inflammatory and metabolic networks shape distinct but related chronic disease states (21–23). In parallel, multi-omics machine-learning models have increasingly combined clinical data with metabolites, proteins or epigenetic markers, yet most approaches rely on early fusion or summary scores and rarely implement explicitly multi-task, multi-class architectures or stacked ensembles that respect the structure of different data blocks (24–26). Moreover, only a few studies have propagated such ML-derived risk signatures into competing-risk frameworks to disentangle how omics-defined risk translates into cause-specific mortality patterns at the population level (27, 28).

Against this backdrop, our study uses the UK Biobank to interrogate how baseline clinical characteristics, inflammation and hematological markers, NMR-based metabolites and Olink-derived proteins jointly define a spectrum of immune-related chronic disease states and their downstream mortality patterns. We classify participants into six mutually exclusive baseline groups—cancer, autoimmune disease, infectious disease, metabolic disease, multiple comorbid conditions and healthy controls—and then apply multi-tower deep learning models and a stacking ensemble to derive integrated, data-driven risk scores for “any chronic disease” as well as multi-class disease status. These ML-derived risk signatures are subsequently embedded into Fine–Gray competing-risk models to quantify their associations with cause-specific mortality, alongside conventional risk factors. By linking multi-omics-informed phenotypes with long-term competing risks of death, this work aims to delineate shared versus disease-specific pathways of immune–metabolic dysregulation, refine population-level risk stratification, and provide a hypothesis-generating and biologically grounded for early prevention and targeted intervention across multiple chronic disease domains.

## 2 Materials and methods

### 2.1 Study design and data sources

**Figure 1:**
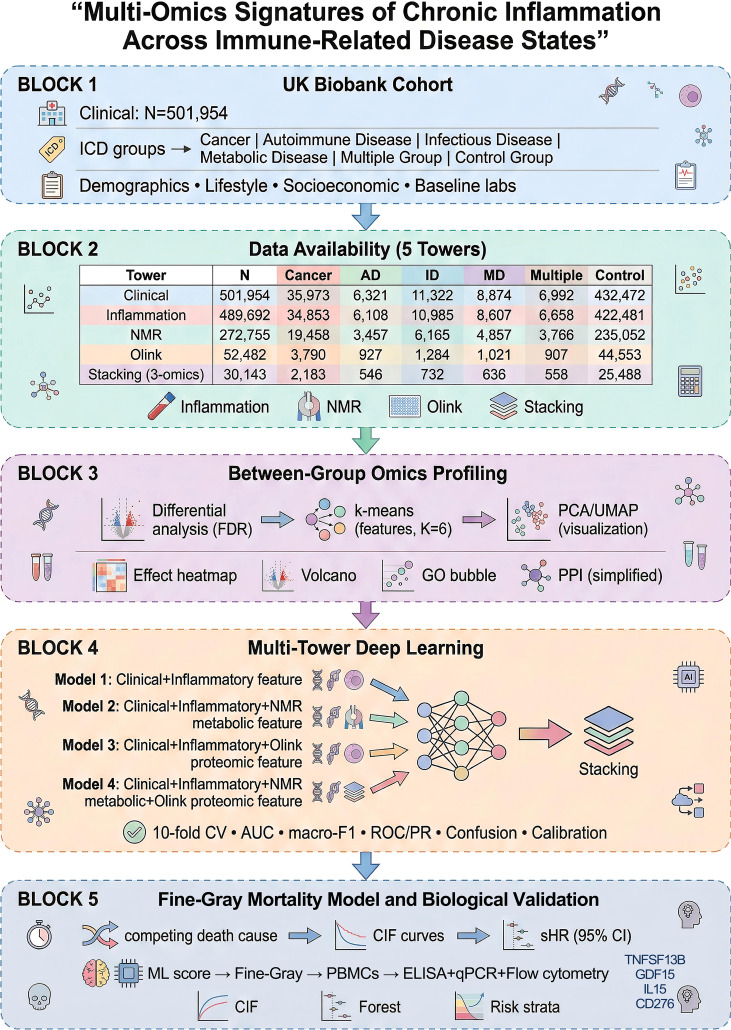
Flowchart of study.

This study employed a cross-sectional and prospective design aimed at exploring the baseline differences in clinical characteristics, inflammation/hematological markers, metabolomics (NMR), and proteomics (Olink) across immune-related disease states, as compared with healthy controls. Participants were classified into six mutually exclusive disease groups using ICD-10 codes recorded prior to baseline. We defined four single-disease categories (Cancer, autoimmune diseases [AD], infectious diseases [ID], and metabolic diseases [MD]). If a participant met the criteria for ≥2 of these categories, they were assigned to the ‘Multiple’ group; otherwise, they were assigned to the corresponding single-disease group. Participants who did not meet any of the four disease-category criteria were assigned to the Control group. A schematic flowchart summarizing the assignment logic is provided in **Figure 1**. Data for this study were sourced from the UK Biobank (29). Data usage and analysis were approved by the UK Biobank, with project ID 194370. The study protocol was ethically reviewed and approved by the UK Biobank ethics committee.

### 2.2 Study variables

The study variables encompass baseline clinical characteristics, inflammation/hematological markers, metabolomics data, proteomics data, and survival outcomes. Initially, the clinical variables include age, sex, ethnicity, educational level, smoking status, alcohol consumption status, sleep duration, physical activity level, and body mass index (BMI). In addition, inflammation/hematological markers include white blood cell count (WBC), hematocrit (HTC), platelet count (PLT), red cell distribution width (RDW), hemoglobin concentration (Hb), C-reactive protein (CRP), among others. The metabolomics data were derived from NMR-based metabolic profiling, with 251 metabolites analyzed, all of which underwent standardization. The NMR metabolomics data were further categorized into 16 official pathways and 7 broader super pathways. This categorization aligns with the hierarchical structure of biological pathways, facilitating a comprehensive understanding of metabolic alterations in various disease states. Proteomics data were obtained using the Olink platform, encompassing over 2,900 proteins, which were also standardized for further analysis. The Olink proteomics measurements are directly mapped to corresponding gene data, providing a strong basis for subsequent gene functional and pathway analyses. The study outcomes include overall survival status, time to death, and cause of death, which will be used in subsequent survival analysis and mortality prediction models.

### 2.3 Disease group comparison

To evaluate the differences across the six disease groups, various statistical and visualization methods were employed. For continuous variables, the Kruskal-Wallis test was performed to identify significant differences among the groups. Pairwise comparisons were subsequently conducted using the Mann-Whitney U test to pinpoint specific group differences. For categorical variables, Pearson’s chi-squared test was applied. Additionally, multiple approaches were utilized to further explore the difference, including heatmaps for the expression patterns of clinical, inflammatory, and omics markers across disease groups. GO enrichment analysis was performed using bubble charts to reveal the biological significance of differential protein expression across groups. Volcano plots were used to visualize the magnitude and significance of metabolic and proteomic differences between disease states, while Sankey diagrams were generated to illustrate the relationships between disease groups, specific proteins, and their associated biological pathways. Furthermore, protein-protein interaction networks were constructed to examine the interactions of key proteins identified in the differential analysis. Lastly, k-means clustering (K = 6) was performed at the feature level to group metabolites or proteins with similar standardized profiles across participants (i.e., clustering rows of the feature × participant matrix). PCA and UMAP were used solely for low-dimensional visualization to illustrate the geometry of feature-level clustering in two dimensions, while disease-group distributions were summarized *post hoc* descriptively.

### 2.4 Machine learning and deep learning models

To assess the predictive value of baseline clinical characteristics, inflammatory/hematological indices, NMR-based metabolomics, and Olink proteomics for the six disease states, we primarily used multi-class deep learning models. Four architectures were specified. Model 1 was a single-tower fully connected network using only clinical and inflammation variables. Models 2 and 3 adopted a two-tower structure in which one tower encoded clinical/inflammatory variables and the other encoded either NMR metabolites (Model 2) or Olink proteins (Model 3). Model 4 was a three-tower network trained in the subset of participants with all three data layers available, combining clinical/inflammatory, NMR, and proteomic representations. Each tower consisted of dense layers with rectified linear unit activation, L2 regularization, batch normalization, and dropout, followed by a shared fully connected block and a 6-node softmax output layer for the six disease categories.

To mitigate class imbalance, inverse-frequency class weights were applied in the categorical cross-entropy loss, and class-specific decision thresholds were tuned on validation data. All models were trained with the Adam optimizer in 10-fold stratified cross-validation, and out-of-fold predicted probabilities were stored. A stacking ensemble was then built using these probabilities (four models × six classes) as meta-features in a multinomial logistic regression meta-learner, yielding final class probabilities and a continuous machine-learning–derived risk score for “any chronic disease” (1 − predicted probability of being in the control group). Model performance was summarized by accuracy, macro-average F1 score, multi-class ROC-AUC (Hand–Till method), and class-specific sensitivity and specificity. For interpretability, surrogate gradient-boosting models were fitted within each data layer and Shapley value–based importance metrics were used to rank clinical, metabolomic, and proteomic features.

### 2.5 Fine-gray competing risks model

To quantify cause-specific mortality risks across disease groups and machine-learning–derived risk strata, we used a Fine–Gray competing risks framework. Cause-specific cumulative incidence functions were first estimated for each type of death in the overall cohort and within each baseline disease group (30). Subsequently, Fine–Gray subdistribution hazard models were fitted separately for each cause of death, treating other causes as competing events. Conventional risk factors included age, sex, body mass index, smoking status, and Townsend deprivation index. In addition, the standardized ML-derived risk scores from the clinical/inflammatory model (ML-risk A), NMR model (ML-risk B), and Olink model (ML-risk C) were incorporated as continuous covariates to capture the aggregated contribution of multi-omics information. Subdistribution hazard ratios (SHR) and 95% confidence intervals were reported for each covariate, and model performance was further examined through assessment of discrimination, calibration, and decision-analytic net benefit for all-cause and cancer-specific mortality.

### 2.6 *In vitro* biological validation of model-identified immune mediators

To biologically ground the model-identified immune-communication signals, we performed an *in vitro* validation focused on four representative mediators highlighted by the stacking model interpretability analyses: GDF15, BAFF (TNFSF13B), IL-15, and the myeloid surface checkpoint CD276. Peripheral blood mononuclear cells (PBMCs) from healthy donors were isolated using density-gradient centrifugation and cultured under standard conditions. Cells were stimulated with canonical inflammatory and polarization cues (Control, IL-4, LPS, IFN-γ, poly(I:C), and LPS+IFN-γ) to emulate distinct immune-activation states relevant to chronic inflammation and immune cell communication. Culture supernatants were collected for ELISA-based quantification of BAFF, GDF15, and IL-15, while matched cellular pellets were harvested for RNA extraction and qPCR quantification of TNFSF13B, GDF15, IL15, and CD276 transcripts. In parallel, surface CD276 expression was assessed by flow cytometry within CD45^+^CD14^+^ monocytes reporting both positivity and distributional shifts. Group comparisons for experimental readouts were conducted using appropriate parametric or non-parametric tests depending on distributional assumptions, with multiplicity controlled for *post-hoc* comparisons.

### 2.7 Statistical methods

All data analyses were conducted in the R (version 4.5.2) programming environment, with statistical analyses and visualizations performed using a comprehensive suite of R packages, including tidyverse, data.table, caret, heatmap, pROC, survival, cmprsk, ggplot2, xgboost, keras3, shapviz, and tensorflow. Continuous variables were expressed as mean ± standard deviation or median (interquartile range), and categorical variables were expressed as counts (percentages). Group comparisons were made using the Kruskal-Wallis test (for continuous variables) and Pearson’s chi-squared test (for categorical variables). Multi-omics differential analysis was performed using linear regression models for metabolites and proteins, with multiple testing correction applied using the Benjamini-Hochberg method. A P-value < 0.05 was considered statistically significant.

## 3 Results

### 3.1 Baseline characteristics

**Figure 2:**
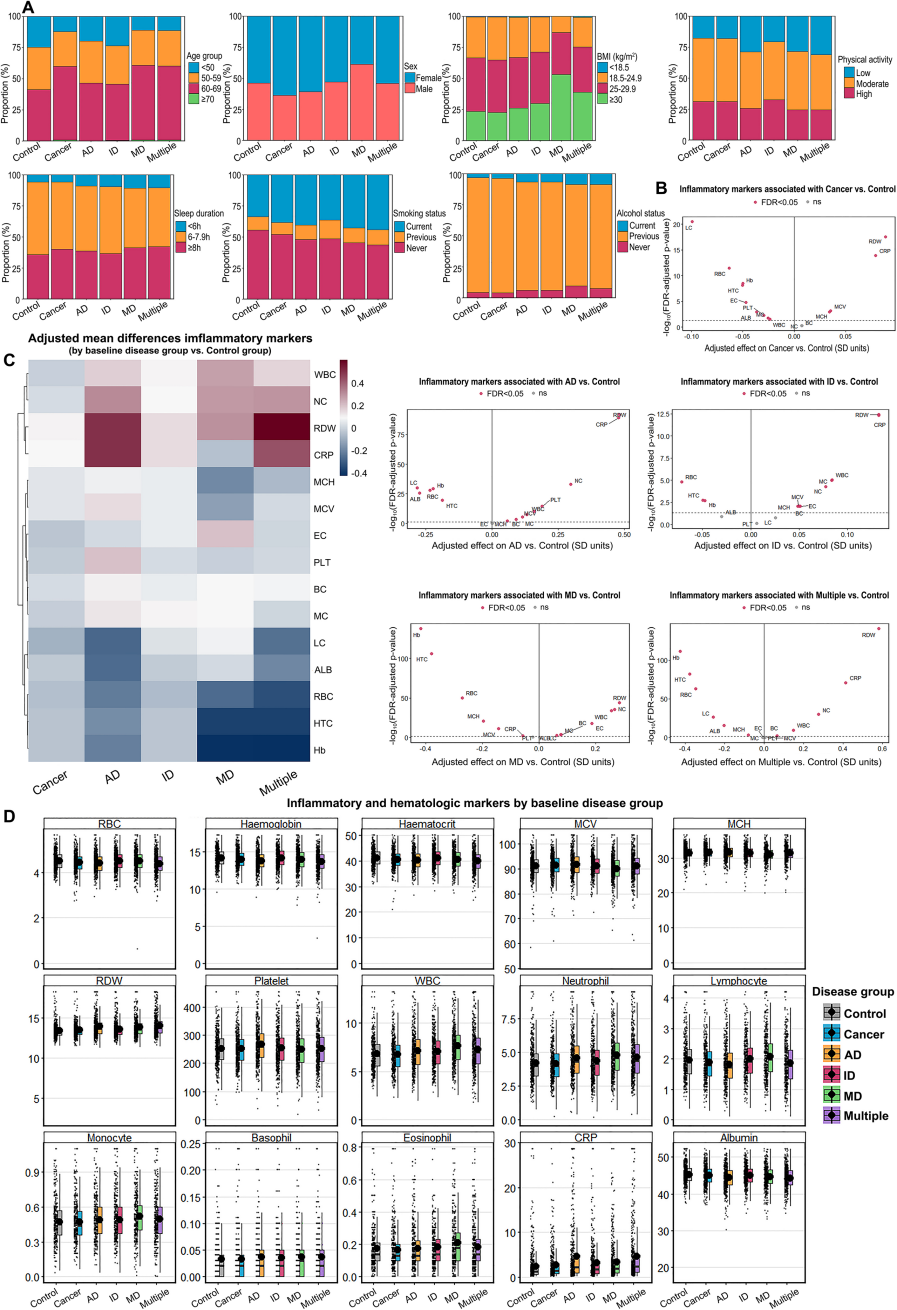
Baseline characteristics and inflammatory markers across disease groups. **(A)** Bar chart representing the proportions of baseline characteristics in Control and five disease groups. **(B)** Heatmap depicting the adjusted mean differences in inflammatory markers between baseline disease groups and the Control group. **(C)** Volcano plots illustrating the adjusted effects of inflammatory markers for each disease group versus Control, showing significant markers with FDR < 0.05. **(D)** Raincloud and boxplots visualizing the distribution of 15 inflammatory and hematological markers across six groups. AD, autoimmune disease; ID Infectious Disease; MD, metabolic disease; CRP, C-reactive protein; WBC, white blood cell count; RDW, red cell distribution width; MCV, mean corpuscular volume; Hb, hemoglobin.

The baseline characteristics of participants across six disease groups were examined, with key demographic and clinical measures displayed in **Table 1**, **Figure 2A**. The demographic distribution showed notable differences across groups in terms of age, sex, and other health indicators. The age at recruitment varied significantly across groups, with Cancer, MD, and Multiple groups having a higher mean age of 60 ± 7 years, compared to the Control and ID groups, which had a mean age of 56 ± 8 years (p < 0.001). Regarding sex, Cancer and AD groups had a higher proportion of females (64% and 61% respectively), while the MD group had a higher proportion of males (61%). For the Townsend deprivation index, the MD group showed a less negative mean of -0.25 ± 3.43, compared with the Control group of -1.33 ± 3.07 (p < 0.001), suggesting relatively higher deprivation in the MD group. The smoking status indicated a high proportion of current smokers in the Cancer and Multiple disease groups (80% and 79% respectively) compared to the Control group (76%), and this difference was statistically significant (p < 0.001). Regarding alcohol consumption, the Control group had the lowest proportion of current drinkers (3.4%), while the other groups, especially MD and Multiple, had higher proportions of current drinkers (9.6% and 9.4% respectively), with significant differences observed (p < 0.001). Physical activity levels also differed significantly between groups, with the MD group showing the highest percentage of individuals with low physical activity (29%), while the Control group had the highest percentage of individuals with high physical activity (31%) (p < 0.001). The Body Mass Index (BMI) was highest in the MD group (31.3 ± 6.1), reflecting higher obesity levels compared to the other groups, with the Control group having a lower BMI (27.3 ± 4.7) (p < 0.001).

**Table 1:** Baseline characteristics and intergroup differences for six diseases group.

Baseline characteristics	Control^1^	Cancer^1^	AD^1^	ID^1^	MD^1^	Multiple^1^	P-value^2^
Age at recruitment, years	56 ± 8	60 ± 7	57 ± 8	57 ± 8	60 ± 7	60 ± 7	<0.001
Sex							<0.001
Female	233,036 (54%)	22,944 (64%)	3,849 (61%)	6,005 (53%)	3,426 (39%)	3,787 (54%)	
Male	199,436 (46%)	13,029 (36%)	2,472 (39%)	5,317 (47%)	5,448 (61%)	3,205 (46%)	
Ethnicity							<0.001
White	390,581 (91%)	33,314 (93%)	5,809 (92%)	9,982 (89%)	7,777 (89%)	6,316 (91%)	
Non-White	39,560 (9.2%)	2,513 (7.0%)	475 (7.6%)	1,241 (11%)	990 (11%)	622 (9.0%)	
Education level							<0.001
Lower than college	163,821 (46%)	13,692 (48%)	2,530 (53%)	4,371 (53%)	3,441 (57%)	2,579 (54%)	
College and/or higher	190,769 (54%)	14,565 (52%)	2,201 (47%)	3,836 (47%)	2,587 (43%)	2,221 (46%)	
Townsend deprivation index	-1.33 ± 3.07	-1.52 ± 2.97	-1.00 ± 3.22	-0.62 ± 3.42	-0.25 ± 3.43	-0.57 ± 3.41	<0.001
Smoking status							<0.001
Current	145,661 (76%)	13,764 (80%)	2,532 (78%)	4,069 (71%)	3,755 (78%)	3,062 (79%)	
Previous	45,239 (24%)	3,413 (20%)	731 (22%)	1,675 (29%)	1,040 (22%)	833 (21%)	
Never	0 (0%)	0 (0%)	0 (0%)	0 (0%)	0 (0%)	0 (0%)	
Alcohol status							<0.001
Current	14,196 (3.4%)	1,339 (3.9%)	432 (7.3%)	743 (7.0%)	764 (9.6%)	606 (9.4%)	
Previous	398,456 (97%)	33,109 (96%)	5,486 (93%)	9,810 (93%)	7,182 (90%)	5,825 (91%)	
Never	0 (0%)	0 (0%)	0 (0%)	0 (0%)	0 (0%)	0 (0%)	
Sleep duration, hours/day	7 ± 1	7 ± 1	7 ± 2	7 ± 2	7 ± 2	7 ± 2	<0.001
Physical activity level							<0.001
Low	59,607 (18%)	4,965 (18%)	1,304 (29%)	1,689 (21%)	1,801 (29%)	1,541 (31%)	
Moderate	170,090 (51%)	13,827 (51%)	2,064 (46%)	3,818 (47%)	2,966 (47%)	2,183 (44%)	
High	103,952 (31%)	8,496 (31%)	1,162 (26%)	2,646 (32%)	1,535 (24%)	1,206 (24%)	
Body mass index, kg/m²	27.3 ± 4.7	27.1 ± 4.7	27.5 ± 5.0	28.1 ± 5.3	31.3 ± 6.1	29.2 ± 6.0	<0.001
Systolic blood pressure, mmHg	138 ± 19	139 ± 19	137 ± 19	137 ± 19	140 ± 18	139 ± 19	<0.001
Diastolic blood pressure, mmHg	82 ± 10	82 ± 10	82 ± 10	82 ± 10	80 ± 10	81 ± 10	<0.001

^1^Mean ± SD; n (%).

^2^Kruskal-Wallis rank sum test; Pearson’s Chi-squared test.

AD, autoimmune disease; ID, infectious disease; MD, metabolic disease; SD, standard difference.

### 3.2 Inflammation and hematological markers comparison

To analyze the differences in inflammation and hematological indicators across six disease groups, heatmap was firstly used in comparing the expression levels of various inflammatory markers between the Control group and the five disease groups (**Figure 2B**). The Cancer group exhibited significantly higher levels of CRP and WBC, indicating a heightened inflammatory state, while the MD and Multiple groups demonstrated lower levels of these markers. Elevated levels of certain inflammatory markers, particularly CRP, were also observed in the AD group. Volcano plots were used to highlight the differential regulation of these markers, emphasizing the magnitude and statistical significance of the differences between each disease group and the Control group (**Figure 2C**). CRP and WBC were consistently upregulated in the Cancer and AD groups, while Hb and MCV showed significant downregulation in the MD and Multiple groups, suggesting possible anemia-related effects in these diseases. Raincloud and boxplots were used to visualize the distribution of 15 inflammatory and hematological markers across the six groups (**Figure 2D**). These plots revealed significant differences in CRP and WBC between the groups, especially between Cancer and Control. The Cancer and AD groups exhibited the highest variability in several markers, while the Control group showed minimal fluctuation, reinforcing its role as a baseline comparator.

### 3.3 Differences in NMR metabolites across disease groups

**Figure 3:**
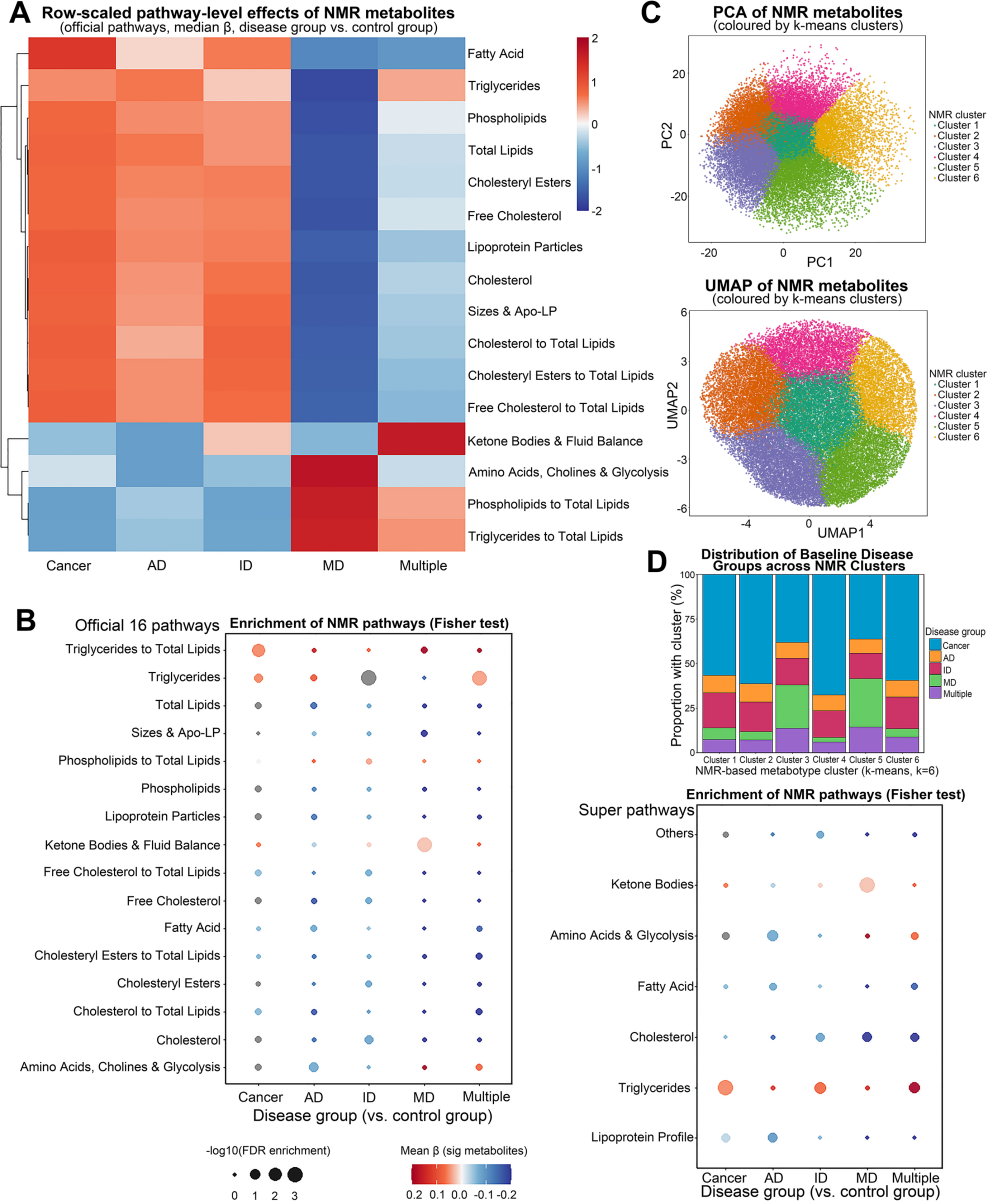
NMR metabolomics differences across disease groups. **(A)** Heatmap of 251 NMR metabolites across 16 official pathways, comparing five disease groups to the Control group. **(B)** Bubble plot showing the enrichment of NMR pathways in each disease group compared to the Control group based on Fisher’s test, for both official 16 pathways and super 7 pathways. **(C)** PCA and UMAP visualization of NMR metabolites features in two dimensions, with metabolites colored by k-means cluster membership (Cluster 1–6). **(D)** Stacked bar chart descriptively summarizing the cluster composition by baseline disease groups as a *post hoc* characterization. NMR, nuclear magnetic resonance; AD, autoimmune disease; ID Infectious Disease; MD, metabolic disease; FDR, false discovery rate; PCA, Principal Component Analysis; UMAP, Uniform Manifold Approximation and Projection.

To investigate the metabolic differences across various disease groups, NMR metabolomics techniques were employed, and multiple analytical methods were utilized. The heatmap depicts the distribution of 251 metabolites across 16 official pathways in the UKB dataset (**Figure 3A**). It is observed that the Cancer group exhibits significantly higher metabolite levels in pathways such as Fatty Acid and Triglycerides, while the Multiple group shows relatively lower levels in these pathways. The AD group demonstrates substantial abnormalities in lipid-related pathways, particularly in Phospholipids and Total Lipids. To further confirm these differences, enrichment analysis results are presented through a bubble plot (**Figure 3B**), highlighting the enrichment of 16 official pathways and 7 super-pathways in different disease groups. Notably, the Cancer and AD groups exhibit significant enrichment in Fatty Acid and Triglycerides pathways. We first applied k-means clustering (K = 6) in the metabolite feature space using the standardized metabolite × participant matrix to group metabolites with similar abundance patterns across participants. PCA and UMAP were then used only to visualize and illustrate the feature-level structure of these clusters in two dimensions (**Figure 3C**). Finally, we descriptively summarized the cluster composition by baseline disease group (**Figure 3D**) to provide a *post hoc* overview of how disease-group–associated metabolite alterations distribute across the six clusters. The Cancer and Multiple groups predominantly occupy Cluster 1 and Cluster 6, while the AD and ID groups are more concentrated in Cluster 2 and Cluster 4.

### 3.4 Differential analysis of Olink-proteomics

**Figure 4:**
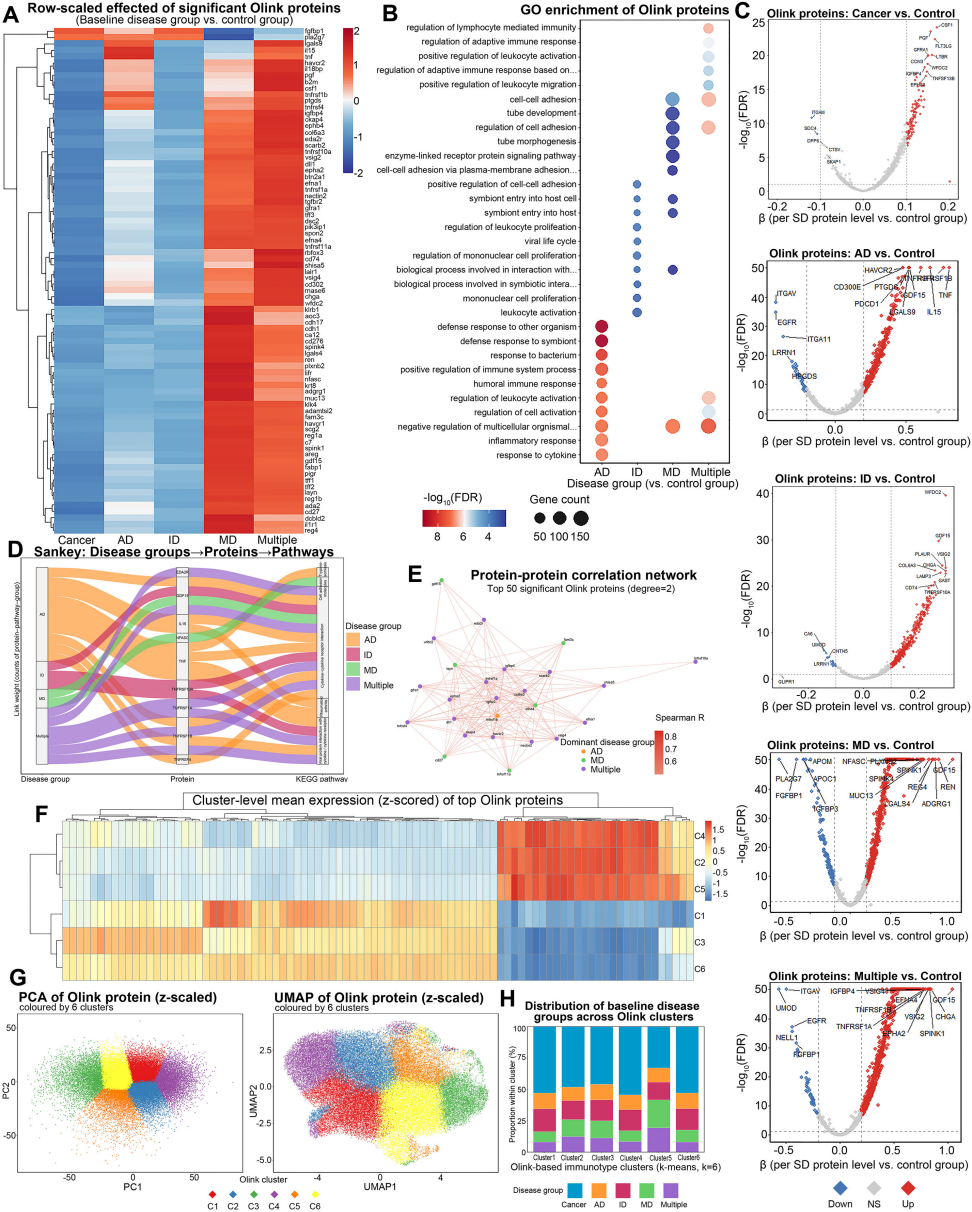
Olink proteomics differences across disease groups. **(A)** Heatmap of the top 80 significant differential proteins (FDR < 0.05) between five disease groups and the Control group. **(B)** Bubble plot showing GO enrichment of differential Olink proteins, with the top 10 enriched pathways displayed for each disease group compared to Control. **(C)** Volcano plots illustrating the adjusted effect of differential Olink proteins for each disease group compared to the Control group. **(D)** Sankey plot showing the relationships between disease groups, top 10 differential Olink proteins, and associated pathways. **(E)** Protein-protein interaction network of the top 50 significant Olink proteins, with the degree of interaction and dominance of disease groups displayed. **(F)** Heatmap summarizing z-scored abundance patterns of proteins grouped into six k-means protein feature clusters (Cluster 1-6). **(G)** PCA and UMAP visualization of z-scored Olink proteins (z-scaled), colored by six identified clusters. **(H)** Stacked bar chart descriptively summarizing the cluster composition by baseline disease group as a *post hoc* characterization. AD, autoimmune disease; ID Infectious Disease; MD, metabolic disease; FDR, false discovery rate; GO, Gene Ontology; FDR, false discovery rate; PCA, Principal Component Analysis; UMAP, Uniform Manifold Approximation and Projection.

To explore the differences between disease groups at the protein level, nearly 3,000 Olink proteomics data measured by the UK Biobank were analyzed. Heatmap was used to display the expression differences of the top 80 differential proteins (FDR < 0.05) between the five disease groups and the Control group (**Figure 4A**). In the Cancer group, several proteins involved in immune response, cell adhesion, and inflammation were significantly upregulated, indicating a heightened inflammatory state. In contrast, these markers were relatively lower in the MD and Multiple disease groups, suggesting a weaker immune response in these groups. The AD group showed elevated levels of specific inflammatory markers. The GO enrichment analysis bubble plot (**Figure 4B**) displayed the functional enrichment of genes associated with these differential proteins. Notably, immune response-related genes were significantly enriched in the Cancer group compared to other groups, while the MD and ID groups showed lower enrichment in immune-related functions. Volcano plots illustrated the differential regulation of proteins between each disease group and the Control group (**Figure 4C**), where the Cancer and AD groups consistently upregulated many differential proteins, while the Multiple group showed relatively smaller differences. The Sankey diagram (**Figure 4D**) showed the relationships between disease groups, differential proteins, and associated pathways, emphasizing the role of immune response and cell adhesion pathways in the Cancer group, particularly associated with proteins such as TNF and CRP. The protein-protein interaction network (**Figure 4E**) revealed strong interactions between proteins related to diseases such as AD and Cancer, particularly in immune and inflammatory pathways, where these proteins formed highly concentrated nodes in the network. Clustering analysis using K-means (k=6) further grouped the differential proteins, and the heatmap (**Figure 4F**) revealed the expression patterns of these proteins across the disease groups. PCA and UMAP (**Figure 4G**) were further used to visualize and illustrate the feature-level clustering structure between clustering and disease groups. Finally, the stacked distribution percentage plot (**Figure 4H**) displayed the distribution of each disease group across different protein clusters, reinforcing the higher proportion of Cancer and AD groups in specific protein clusters.

### 3.5 Multi-omics deep learning and stacking ensemble model

**Figure 5:**
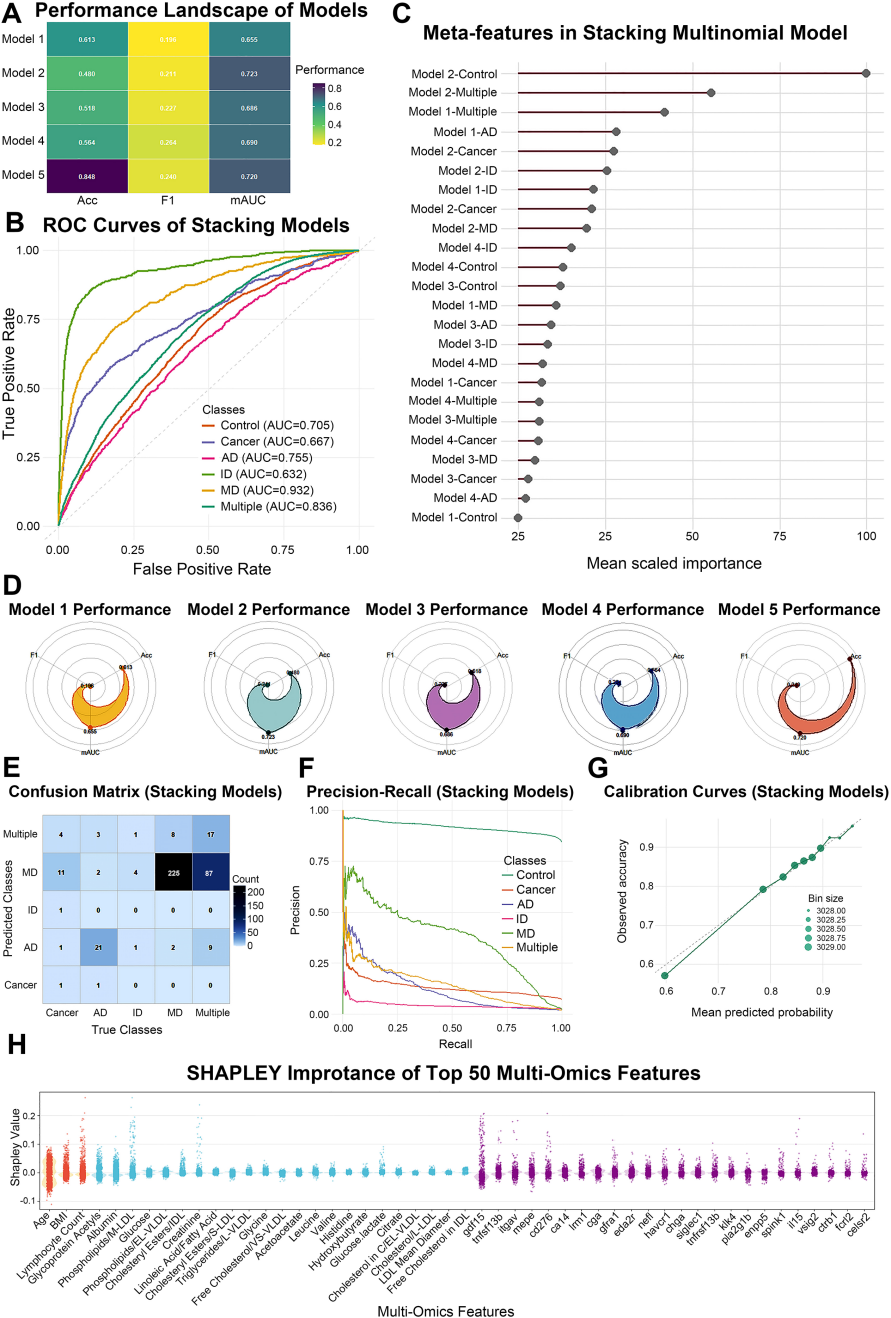
Performance of deep learning and stacking models for multi-disease classification. **(A)** Heatmap showing accuracy, macro-average F1, and multi-class AUC (mAUC) for five models: Model 1 (clinical + inflammatory markers), Model 2 (clinical + inflammatory markers + NMR metabolites), Model 3 (clinical + inflammatory markers + Olink proteins), Model 4 (three-tower multi-omics model), and Model 5 (stacking meta-model integrating Models 1–4). **(B)** One-versus-rest ROC curves of the stacking meta-model across six outcome classes, with AUC values indicated in the legend. **(C)** Scaled importance of base-model class-specific probabilities used as meta-features in the multinomial stacking model. **(D)** Radar plots summarizing accuracy, macro-F1, and mAUC for each individual model. **(E)** Normalized confusion matrix of the stacking model showing predicted versus true classes. **(F)** Precision–recall curves of the stacking model for each disease class. **(G)** Calibration curve of the stacking model comparing mean predicted probability with observed accuracy across deciles of predicted risk. **(H)** SHAP beeswarm plot displaying the contribution of the top 50 multi-omics features to the stacking model risk prediction, colored by data type (clinical/inflammatory, NMR metabolites, and Olink proteins). AD, autoimmune disease; ID, infectious disease; MD, metabolic disease; mAUC, multi-class area under the ROC curve; PR, precision–recall; SHAP, Shapley additive explanations; NMR, nuclear magnetic resonance.

To comprehensively evaluate disease-state classification, we constructed five deep learning–based models: a clinical plus inflammatory model (Model 1), a clinical+inflammation+NMR model (Model 2), a clinical+inflammation+Olink proteomics model (Model 3), a three-tower multi-omics network (Model 4), and a stacking meta-model (Model 5) that takes the out-of-fold predicted probabilities from the four base models as inputs. Model comparison was based on 10-fold cross-validated out-of-fold predictions (**Figure 5**). The performance landscape showed that Model 1 provided a basic level of discrimination but was inferior to the omics-augmented models in terms of accuracy, macro-average F1, and multi-class AUC. Adding NMR or Olink data (Models 2 and 3) substantially improved overall performance, with the NMR-based model yielding the highest macro-AUC (0.723) among single-block models. The three-tower multi-omics model (Model 4) showed slightly less stable performance, likely due to reduced sample size after requiring complete multi-omics data. In contrast, the stacking model (Model 5) achieved the best accuracy (0.848), macro-F1 (0.240), and mAUC (0.720) across all configurations, indicating that integrating heterogeneous information at the meta-learning level effectively combines clinical, metabolic, and proteomic signals (**Figures 5A, D**).

Class-specific ROC curves for the stacking model demonstrated overall improvement over the baseline model for MD and Multiple disease groups, with stable AUCs for AD and Cancer and high specificity for the Control group (**Figure 5B**). The confusion matrix further showed that most individuals were correctly classified, such as AD versus ID and MD versus Multiple (**Figure 5E**). Precision–Recall curves and calibration analysis indicated that the stacking model maintained favourable precision and recall in the medium-to-high risk range, with predicted probabilities closely aligned with observed risks and only mild underestimation at the extreme high-risk tail (**Figures 5F, G**). Within the second-layer multinomial meta-model, the contributions of base-model outputs were heterogeneous. Meta-feature importance revealed that the NMR model probabilities for Multiple and Control, together with the three-tower model outputs for MD and Cancer, carried the largest weights, suggesting that these signals act as key anchors in the final decision (**Figure 5C**). Shapley-value–based interpretation of the stacking-derived risk score identified the top 50 contributing features across clinical/inflammatory markers, NMR metabolites, and Olink proteins. Age, BMI, CRP, and several blood cell indices were among the leading clinical and inflammatory contributors; lipoprotein and lipid-related NMR metabolites (multiple HDL, LDL, and triglyceride fractions) played major roles in differentiating disease patterns; and proteins such as GDF15, CD276, and TNFSF13B, involved in immune and inflammatory pathways, ranked highly among proteomic predictors (**Figure 5H**). Together, these findings indicate that multi-omics deep learning combined with stacking not only improves multi-class classification performance but also delineates the layered contributions of diverse biomarkers, providing a quantitative basis for subsequent mechanistic analyses and risk stratification.

### 3.6 Fine–gray models and ML-derived risk scores

**Figure 6:**
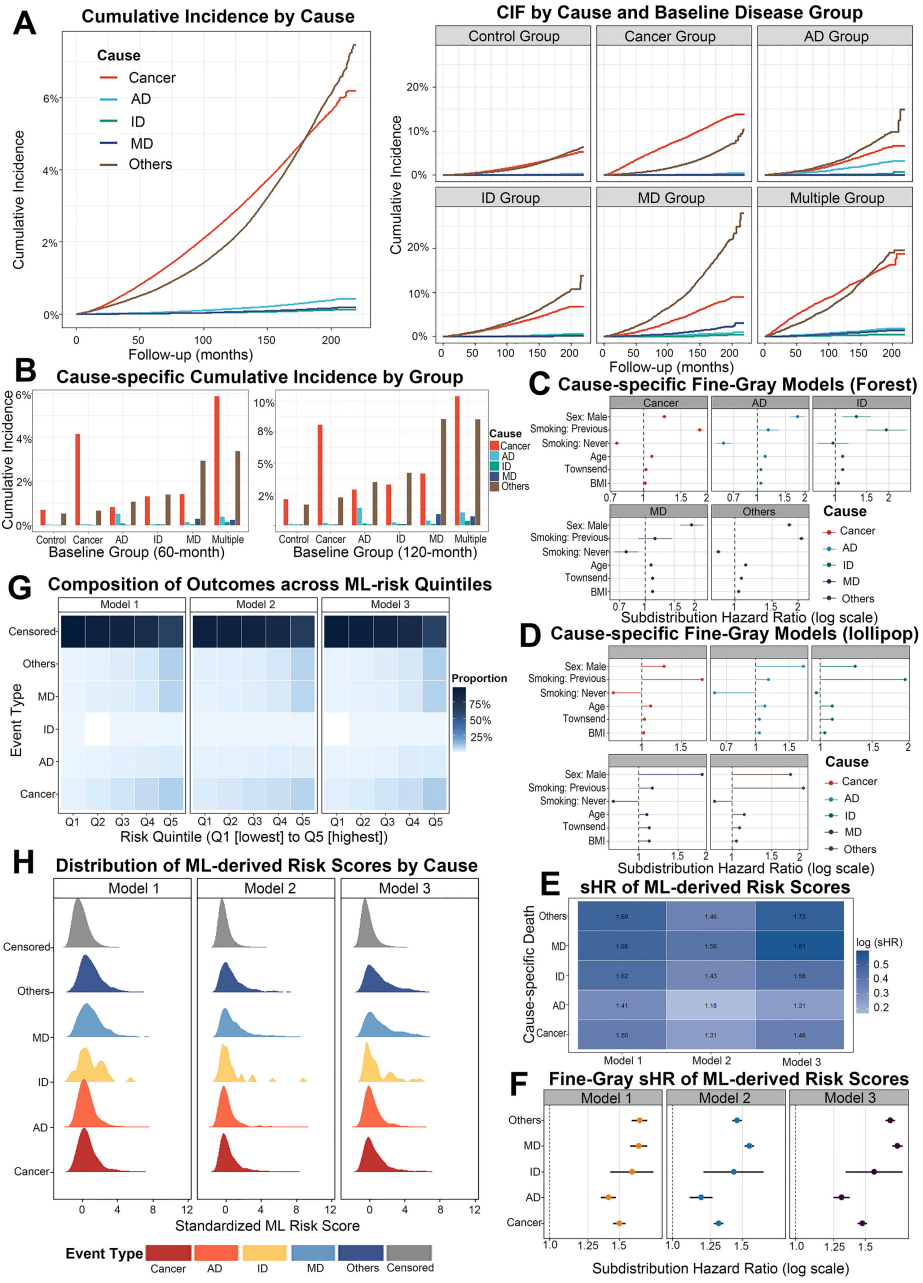
Cause-specific competing risks and integration of ML-derived risk scores. **(A)** Left: overall cumulative incidence functions for cancer, AD, ID, MD and other-cause death. Right: cause-specific cumulative incidence functions stratified by baseline disease group (Control, Cancer, AD, ID, MD, Multiple). **(B)** Bar plots showing 60-month and 120-month cause-specific cumulative incidence by baseline disease group. **(C)** Cause-specific Fine–Gray models including age, sex, BMI, smoking status, and Townsend index, presented as forest plots of subdistribution hazard ratios (sHR) on a log scale for each cause of death. **(D)** Alternative lollipop representation of the same cause-specific Fine–Gray models, highlighting the direction and magnitude of associations across causes. **(E)** Heatmap of sHRs for machine-learning–derived risk scores from Models 1–3 across different causes of death. **(F)** Forest plot of Fine–Gray sHRs for ML-derived risk scores, summarizing their effects on cancer, AD, ID, MD and other-cause mortality. **(G)** Heatmaps showing the composition of outcomes (cancer death, AD death, ID death, MD death, other-cause death, censoring) across quintiles of ML risk scores from Models 1–3. **(H)** Ridge density plots of standardized ML-derived risk scores by event type for each model, illustrating separation of risk distributions between survivors and different causes of death. AD, autoimmune disease; ID, infectious disease; MD, metabolic disease; CIF, cumulative incidence function; sHR, subdistribution hazard ratio; ML, machine learning.

To characterize long-term cause-specific mortality, we applied Fine–Gray competing risks models. In the overall cohort, the cumulative incidence of cancer-related death increased rapidly early during follow-up and remained the highest across the entire period, whereas AD-, ID-, and MD-related deaths showed much lower cumulative risks (**Figure 6A**). At fixed time points of 60 and 120 months, cause-specific cumulative incidences further confirmed that Cancer and Multiple groups had the greatest burden of cancer mortality, whereas the MD group showed progressively increased risks of metabolic/cardiovascular and other-cause deaths (**Figure 6B**). In Fine–Gray models including only conventional risk factors, older age, former or current smoking and higher deprivation were associated with increased subdistribution hazard for several causes of death, while higher BMI showed neutral or mildly protective effects for some outcomes (**Figures 6C, D**). We then incorporated the standardized multi-disease risk scores derived from deep-learning Models 1–3 as composite covariates. Outcome composition across quintiles of each ML risk score showed that, from Q1 to Q5, the proportions of cancer and other-cause deaths increased monotonically, whereas the proportion of censored individuals decreased, indicating that ML-derived scores effectively stratified long-term mortality risk (**Figure 6G**). Cause-specific distributions of the scores further demonstrated that individuals who died from cancer consistently had the highest ML risk across all three models, followed by AD and MD deaths, whereas censored and non-fatal events clustered in the low-risk range (**Figure 6H**). In extended Fine–Gray models including the ML risk scores, all three models showed robust positive associations with multiple causes of death, with subdistribution hazard ratios largely between 1.3 and 1.8 and the strongest effects for cancer and other-cause mortality (**Figures 6E, F**). These findings suggest that deep-learning–integrated multi-omics signatures provide additional prognostic value for long-term survival and the distribution of causes of death beyond traditional risk factors.

### 3.7 Experimental validation of model-identified mediators

**Figure 7:**
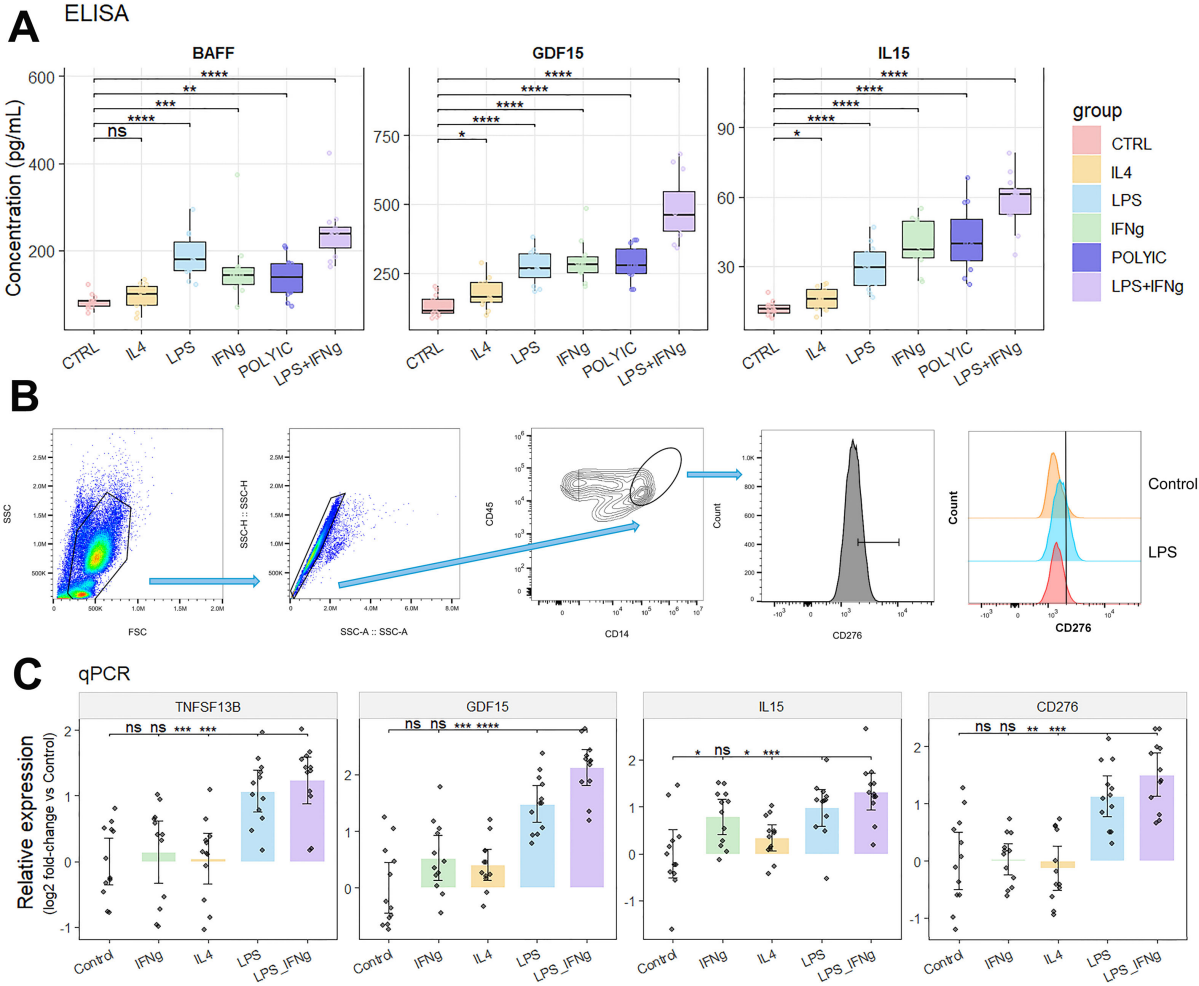
*In vitro* biological validation of model-prioritized immune mediators. **(A)** ELISA quantification of BAFF (TNFSF13B), GDF15, and IL-15 in culture supernatants under indicated stimulation conditions. **(B)** Flow cytometry gating strategy and representative overlays showing increased CD276 expression on CD45^+^CD14^+^ monocytes following LPS stimulation relative to control. **(C)** qPCR validation of TNFSF13B, GDF15, IL15, and CD276 transcripts under indicated stimulation conditions, reported as relative expression versus control. PBMC, peripheral blood mononuclear cell; IFN-γ, interferon gamma. *: p<0.05, **: p<0.01, ***: p<0.001, ****: p<0.0001, ns: p>0.05.

To biologically validate key mediators highlighted by the multi-omics stacking framework, we stimulated PBMC cultures with inflammatory cues and quantified BAFF, GDF15, and IL-15 at the protein level and TNFSF13B, GDF15, IL15, and CD276 at the transcript level (**Figures 7A, C**). Across conditions, LPS-based stimulation produced the most consistent induction of all three secreted mediators, with the combined LPS+IFN-γ condition yielding the highest concentrations for BAFF, GDF15, and IL-15 (**Figure 7A**). Poly(I:C) and IFN-γ alone showed intermediate upregulation patterns, whereas IL-4 exerted comparatively modest effects. Concordantly, qPCR measurements demonstrated significant increases of TNFSF13B, GDF15, and IL15 transcripts under LPS and LPS+IFN-γ stimulation, mirroring the protein-level directionality (**Figure 7C**). For the surface immune checkpoint CD276, flow cytometry within CD45^+^CD14^+^ monocytes revealed a clear rightward shift of CD276 signal following LPS exposure relative to control, indicating increased surface expression at the myeloid compartment (**Figure 7B**). Consistent with these flow findings, CD276 transcript levels were also elevated in LPS-driven conditions, with the strongest induction observed in the LPS+IFN-γ group (**Figure 7C**).

## 4 Discussion

In this large, deeply phenotyped UK Biobank cohort, we integrated clinical characteristics, inflammation and hematological indices, NMR-based metabolomics, and Olink proteomics to delineate an immune-related multi-disease spectrum and to build data-driven risk–prediction models across six chronic disease states. Our findings show that systemic inflammation, lipid and amino acid metabolism, and immune-related proteomic signals jointly shape disease-specific and shared patterns, and that multi-omics deep learning combined with stacking and competing-risks modelling can translate these patterns into clinically interpretable risk strata for both disease classification and long-term cause-specific mortality.

Our multi-layer analyses highlight pronounced heterogeneity of inflammatory, metabolic, and proteomic profiles across the immune-related disease spectrum, extending prior evidence from single-disease or organ-specific cohorts. Deng et al. recently reported that diverse cardiometabolic, inflammatory, and malignant phenotypes share partially overlapping but disease-specific protein signatures, particularly within immune and vascular pathways (7). Similar large-scale proteomic efforts have demonstrated that thousands of circulating proteins are systematically associated with cardiometabolic, autoimmune, and oncologic outcomes, underscoring the concept of a shared “immune–metabolic hub” linking chronic inflammation to multimorbidity (31–33). In our study, cancer and autoimmune disease groups were characterized by consistently elevated CRP, WBC, lipid-related metabolites, and inflammation-related proteins such as GDF15 and CD276, whereas metabolic and multiple-disease groups showed more complex patterns with coexisting inflammatory activation and hematologic perturbations (34–36). These observations echo recent multi-omics work showing that multimorbidity clusters and disease trajectories are strongly shaped by systemic inflammatory tone and lipid metabolism, but we additionally reveal that distinct combinations of NMR-defined lipid subclasses and proteomic immune markers help differentiate cancer-, autoimmune-, infection-, and metabolic-dominant states within a unified framework (37–40).

From a modelling perspective, our multi-tower deep learning and stacking framework illustrates how heterogeneous information from clinical, NMR, and proteomic layers can be efficiently fused to improve multi-disease classification. Conventional machine learning models and single-modality deep networks have achieved promising performance in predicting individual diseases from electronic health records or omics panels, but they often treat each outcome in isolation and underutilize cross-disease structure (41–43). Recent works have advocated multi-task or multi-modal architectures for joint disease prediction, yet most applications remain limited to pairs of related phenotypes or to small sample sizes (44–46). Our stacking framework yielded an overall improvement over the baseline model and demonstrated stronger class-wise performance in MD/Multiple/Control based on per-class ROC/PR and confusion-matrix analyses. Notably, macro-F1 is the unweighted average of per-class F1 scores and therefore can be conservative under class imbalance, as it penalizes underperforming minority or harder-to-separate classes; thus, it should be interpreted alongside class-specific metrics. This pattern is consistent with recent multi-omics integration studies, where ensemble or hierarchical architectures were shown to outperform any single data block, particularly for complex, overlapping phenotypes such as cardiometabolic–inflammatory multimorbidity (24, 47, 48). Importantly, by combining tower-specific Shapley value analysis with the meta-learner, we were able to decompose the contribution of clinical, metabolic, and proteomic signals to each disease class, providing a transparent link between abstract deep-learning features and interpretable biomarkers that is often missing in earlier deep learning applications.

Machine learning–derived risk scores in this study extended the cross-sectional disease classification into a longitudinal framework of cause-specific mortality. Across all three ML risk scores, we observed a clear monotonic gradient in the cumulative incidence of cancer and other-cause death from the lowest to the highest risk quintiles, and these associations remained robust after adjustment for age, sex, adiposity, smoking, and deprivation in Fine–Gray models. This pattern is consistent with the growing literature showing that data-driven risk scores derived from high-dimensional clinical or omics data can capture multi-system vulnerability and improve prediction of all-cause and cause-specific mortality beyond traditional risk factors (34, 49–51). Recent works have emphasized that modern survival models, including penalized regression, tree-based ensembles, and deep neural networks, are particularly suited to modeling complex, competing-risks endpoints in large cohorts (52–54). Our results complement these advances by demonstrating that ML-derived risk scores summarizing chronic disease burden at baseline are strongly and consistently associated with both cancer and non-cancer death, suggesting that latent multi-organ dysfunction and immune-metabolic dysregulation, as encoded in the risk scores, translate into long-term excess mortality risk.

Our work is also closely aligned with, but extends beyond, prior multi-omics and machine-learning studies that have largely focused on single cancer types or organ-specific outcomes. Gillette et al. summarized how integrating genomics, transcriptomics, and proteomics with ML algorithms can refine lung cancer prognostication, yet most existing models were disease-specific and built within relatively narrow clinical contexts (10). Recent cancer-oriented frameworks, such as stacked multi-omics fusion networks for survival in breast cancer, have shown that hierarchical or stacked architectures can outperform single-modality models by capturing complementary information across data layers (17, 21, 55, 56). In parallel, proteogenomic consortia have demonstrated that integrating proteomics with genomic and transcriptomic data can reveal clinically meaningful subtypes and therapeutic vulnerabilities in hepatocellular carcinoma and other solid tumors, underscoring the biological value of multi-layer data integration (57). Complementing these disease-specific efforts, methodological reviews in high-dimensional survival analysis and AI-driven precision oncology have emphasized that model architectures, regularization strategies, and ensemble schemes critically shape performance and generalizability in multi-omics prediction tasks. Against this backdrop, our study differs in two key aspects: first, it operationalizes a unified multi-disease framework spanning cancer, autoimmune, infectious, metabolic, and multimorbid states rather than a single index disease; second, it combines tower-based deep learning with stacking at the probability level to derive a generic “chronic disease burden” score that remains prognostically informative for multiple competing causes of death. Together, these extensions position our work as a bridge between organ-specific multi-omics modeling and population-level, cross-disease risk stratification.

A central concern for population-scale computational omics is whether the learned signatures reflect biologically inducible programs rather than cohort-specific artifacts. We therefore added an orthogonal *in vitro* validation layer targeting four interpretable mediators (GDF15, BAFF/TNFSF13B, IL-15, and CD276) prioritized by the stacking model. Under canonical inflammatory activation, particularly LPS and LPS+IFN-γ, we observed coordinated induction at both secreted-protein and transcript levels, together with increased surface CD276 on CD14^+^ myeloid cells. This pattern is consistent with the view that chronic inflammation is not a uniform “common soil,” but an ensemble of stimulus-conditioned communication states in which myeloid compartments act as amplifiers and translators of systemic cues (58–63). Notably, recent plasma-proteome resources from the UK Biobank have demonstrated that inflammatory and immune-regulatory proteins encode reproducible, trait-linked axes at population scale, enabling mechanistic hypothesis generation from observational proteomics (7). Our validation extends this paradigm by showing that selected, model-prioritized mediators are experimentally inducible in a myeloid-inflammatory context, thereby strengthening the evidentiary bridge between computational stratification and immune cell communication biology.

Several strengths and limitations of our work merit consideration. Leveraging the scale and depth of the UK Biobank, we jointly analyzed clinical phenotypes, inflammation-related hematological markers, NMR metabolomics, and Olink proteomics within a harmonized disease spectrum, and combined tower-based deep learning, stacking ensembles, and competing-risks regression in a single analytic pipeline. This design allowed us to move from descriptive cross-sectional comparisons to multi-class disease recognition and finally to cause-specific mortality prediction within one coherent framework. Nonetheless, key caveats remain. First, although UK Biobank offers unparalleled sample size and data richness, its volunteer nature and predominantly European ancestry limit immediate generalizability to more diverse populations. Second, despite extensive adjustment, residual confounding and indication bias cannot be fully excluded, particularly for participants with complex multimorbidity. Third, the deep learning and stacking models (multi-tower architecture reduces effective sample size and event counts), while relatively stable in cross-validation, were trained and evaluated within a single cohort. Given the UK Biobank’s relatively constrained geographic setting and the comparatively concentrated recruitment period, a geographic- or time-split validation would be expected to offer limited incremental benefit while further reducing the number of minority-class samples in the held-out set. Therefore, we used stratified 10-fold cross-validation and reported multiple complementary metrics (e.g., per-class ROC/PR, confusion matrices, calibration) to comprehensively assess model performance. External validation in independent biobanks or healthcare systems will be essential before clinical deployment. Future work should therefore prioritize: validating and recalibrating the ML risk scores in ethnically and clinically diverse cohorts; integrating additional data layers such as imaging, longitudinal trajectories, and genomics to further dissect mechanisms; and embedding these models in decision-support tools that can dynamically inform prevention, surveillance, and treatment strategies.

## 5 Conclusions

In this large UK Biobank cohort, we used multi-omics deep learning and competing-risk modelling to decode how systemic inflammatory, metabolic and proteomic signatures jointly organize across cancer, autoimmune, infectious and metabolic diseases. By integrating clinical and hematological indices with NMR metabolites and Olink immune-related proteins in multi-tower networks and a stacking ensemble, we derived machine-learning risk scores that capture shared axes of chronic inflammation and immune communication across disease states. These scores were strongly and independently associated with cancer-related and other cause-specific mortality beyond traditional risk factors, and were driven by coherent clusters of cytokines, myeloid and lymphoid markers, and lipid-related metabolites. Importantly, we complemented population-scale computation with *in vitro* biological validation: inflammatory stimulation of healthy-donor PBMCs induced BAFF/TNFSF13B, GDF15 and IL-15 secretion and transcription, and increased CD276 on CD14^+^ monocytes, supporting the myeloid inflammatory inducibility of model-highlighted mediators. Together, our findings outline a population-scale map of chronic immune–metabolic communication that links disease clustering with long-term outcomes and may guide future mechanism-oriented and precision immunomodulatory strategies.

## Data Availability

The original contributions presented in the study are included in the article/supplementary material. Further inquiries can be directed to the corresponding author/s.
